# Hybrid *Vitis* Cultivars with American or Asian Ancestries Show Higher Tolerance towards Grapevine Trunk Diseases

**DOI:** 10.3390/plants12122328

**Published:** 2023-06-15

**Authors:** András Csótó, Antal Nagy, Nóra Laurinyecz, Zóra Annamária Nagy, Csaba Németh, Erzsébet Krisztina Németh, Anna Csikász-Krizsics, Nándor Rakonczás, Florence Fontaine, Erzsébet Fekete, Michel Flipphi, Levente Karaffa, Erzsébet Sándor

**Affiliations:** 1 Institute of Plant Protection, Faculty of Agricultural and Food Science and Environmental Management, University of Debrecen, H-4032 Debrecen, Hungary; csoto.andras@agr.unideb.hu (A.C.); nagyanti@agr.unideb.hu (A.N.); laurinyecznora14@gmail.com (N.L.); 2 Kálmán Kerpely Doctoral School, University of Debrecen, H-4032 Debrecen, Hungary; 3 Research Institute for Viticulture and Oenology Badacsony, Hungarian University of Agriculture and Life Sciences, H-8263 Badacsonytomaj, Hungary; zoranagy@gmail.com (Z.A.N.); nemeth.csaba@uni-mate.hu (C.N.); 4 Research Institute for Viticulture and Oenology Kecskemét, Hungarian University of Agriculture and Life Sciences, H-6000 Kecskemét, Hungary; nemeth.erzsebet.krisztina@uni-mate.hu; 5 Research Institute for Viticulture and Oenology, University of Pécs, H-7634 Pécs, Hungary; krizsics.anna@pte.hu; 6 Institute of Horticulture, Faculty of Agricultural and Food Science and Environmental Management, University of Debrecen, H-4032 Debrecen, Hungary; rakonczas@agr.unideb.hu; 7 Unité Résistance Induite et Bioprotection des Plantes, USC INRAE 1488, URCA, Université de Reims Champagne-Ardenne, 51687 Reims, France; florence.fontaine@univ-reims.fr; 8 Department of Biochemical Engineering, Faculty of Science and Technology, University of Debrecen, H-4032 Debrecen, Hungary; kicsizsoka@yahoo.com (E.F.); drir.michelflipphi@gmail.com (M.F.); levente.karaffa@science.unideb.hu (L.K.); 9 Institute of Food Science, Faculty of Agricultural and Food Science and Environmental Management, University of Debrecen, H-4032 Debrecen, Hungary

**Keywords:** interspecific cultivars, *Vitis vinifera*, *Vitis amurensis*, *Vitis rupestris*, *Vitis labrusca*, grape germplasm collection, GTDs

## Abstract

Grape production worldwide is increasingly threatened by grapevine trunk diseases (GTDs). No grapevine cultivar is known to be entirely resistant to GTDs, but susceptibility varies greatly. To quantify these differences, four Hungarian grape germplasm collections containing 305 different cultivars were surveyed to determine the ratios of GTDs based on symptom expression and the proportion of plant loss within all GTD symptoms. The cultivars of monophyletic *Vitis vinifera* L. origin were amongst the most sensitive ones, and their sensitivity was significantly (*p* < 0.01) higher than that of the interspecific (hybrid) cultivars assessed, which are defined by the presence of *Vitis* species other than *V. vinifera* (e.g., *V. labrusca* L., *V. rupestris* Scheele, and *V. amurensis* Rupr.) in their pedigree. We conclude that the ancestral diversity of grapes confers a higher degree of resilience against GTDs.

## 1. Introduction

Grapevine trunk diseases (GTDs) are among the most important diseases of grapevines, with estimated losses of 1.5 billion USD worldwide, while the average GTD incidences were reported to be between 10% (Spain) and 22% (Italy) in European vineyards [1,2,3,4]. Moreover, an increase in disease incidence has been recognized in several grape growing countries such as Spain, Italy, and Canada [5,6,7,8]. GTD fungal pathogens colonize the woody part of the plant, producing different toxins and enzymes, resulting leaf symptoms (tiger stripes), stunted growth, reduced quantity and quality of grape, and dieback of the plant [2]. GTDs are complex diseases, including esca, eutypa dieback, black foot, Botryosphaeria, and Petri diseases, and are affected by several biotic and abiotic factors [2,4,9,10,11,12]. More than 100 fungal species have been recognized as GTD pathogens, characterized by different taxonomic statuses, disease cycles, fungicide sensitivity, and host ranges [13]. Moreover, infections do not usually manifest rapidly, and can linger on for years. Factors and circumstances that turn the latent infection into an active one, giving rise to mild (e.g., foliar symptoms) or serious symptoms (partial or whole plant dieback), are not fully understood. Wounds, environmental stress (frost, drought, flood), and increased age of vineyards appear to correlate with increased disease incidence of GTDs [4,14,15,16,17]. Chronic symptom expression does not necessarily lead to significant yield or quality loss of the fruit or plant within a few years [4,18]; conversely, apoplexy of the trunk leads to plant loss and results in irreversible economic loss in the plantation. Replanting vineyards with young, healthy vines is challenging and often unsuccessful.

Tolerant plant cultivars are widely used as they are one of the most effective means of controlling plant diseases, providing economic and environmentally friendly plant protection technology while reducing pesticide usage and dependency [19]. Disease-resistant cultivars would also provide solutions when effective protection by chemical pesticides is not available, as in the case of GTDs [20,21,22].

Due to the susceptibility of traditional European grape varieties to different pathogens, an interspecific hybrid breeding program was started in France in the early forties of the 19th century, by crossing *Vitis vinifera* varieties from France with American species, which resulted in more resistant, high-quality hybrids that exhibited partial resistance towards fungal pathogens [23]. The hybrid offspring were subsequently used in resistance breeding programs in Hungary as ‘Seibel’ and ‘Seyve-Villard’ varieties [24]. However, with the propagating plants, the phylloxera (*Daktulosphaira vitifoliae* Fitch) insect pest was also introduced, which resulted in a dramatic loss of plants in European vineyards. It also has become common practice to graft American rootstock, resistant to phylloxera, to preserve susceptible cultivated European varieties, and grape breeding programs were initiated to control phylloxera, powdery mildew (*Erysiphe necator* Schwein.), and downy mildew (*Plasmopara viticola* (Berk. et Curt.) Berl. et De Toni) [23,25,26,27,28].

*Vitis amurensis* Rupr., native to China, has several beneficial properties, such as cold resistance and resistance against several phytopathogens causing diseases, such as grape crown gall (*Allorhizobium vitis*), white rot (*Coniella diplodiella* (Speg.) Petr. Et Syd.), downy mildew, and anthracnose of grapes (*Elsinoe ampelina* Shear). Therefore, it is often used as rootstock or in breeding interspecific hybrids [29,30,31,32,33,34,35,36]. The introduction of American and Asian grape species into the breeding programs increases genetic diversity and compensates for the bottleneck effect (when the size of a population is severely reduced), which has developed historically as a consequence of the domestication of *V. vinifera* [37,38].

There are no *V. vinifera* cultivars known to be completely resistant to GTD pathogens; however, considerable differences in sensitivity have been recognized during in planta tests and in field surveys (Table 1). Differences were observed between the tolerance to different GTD pathogenic fungi in one cultivar, which may be due to the various climate conditions and/or grape-producing technologies. In the case of eutypa dieback, Dubos [39] categorized Aligote, Grolleau, Merlot, Semillion, and Sylvaner cultivars as resistant, and later Carter [40] reported possible resistance against *Eutypa lata* (Pers.) Tul. & C. Tul in some French cultivars. Borgo et al. [41] and Murolo-Romanazzi [42] classified the degree of GTD expression for six and 86 varieties, distinguishing between red and white grape varieties. Sosnowski et al. [43] ranked 118 varieties based on plant death and foliar symptoms. These and other studies have shown that, among internationally recognized and cultivated varieties, Cabernet Sauvignon, Cabernet Franc, and Sauvignon Blanc are particularly susceptible to GTDs, while Merlot is much more resilient.

Both GTD chronic symptom expression and apoplexy combined with subsequent loss of plants were monitored in four Hungarian grape germplasm collections containing a total of 305 different cultivars. Disease incidence (DI) was calculated to compare (i) the degree of GTD sensitivity of the most important international and national grape cultivars, and (ii) the severity of GTD symptoms in cultivars with monophyletic *V. vinifera* origin and interspecific (hybrid) cultivars with various American or Asian *Vitis* species in their pedigree. These data may provide important information for extended and future grape breeding programs.

## 2. Results

**Figure 1:**
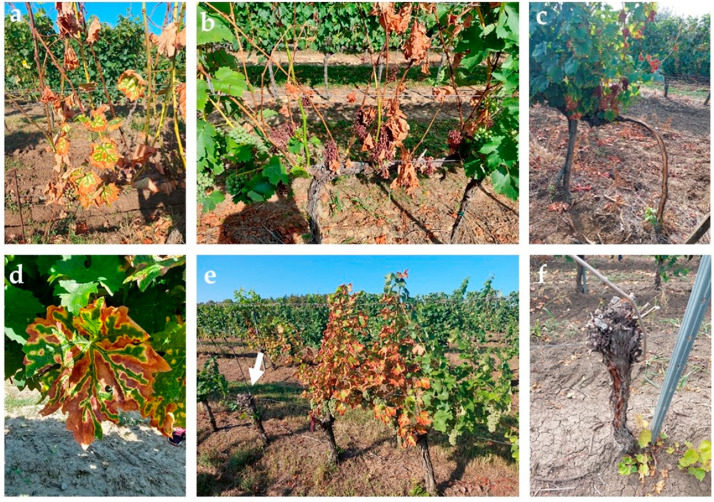
GTDs symptoms: (**a**,**d**) leaf stripes; (**b**) partial dieback; (**c**) esca symptoms with white rot and leaf stripe; (**e**) dead plant from previous dieback (indicated by arrow) and new (annual) symptomatic plants (middle and right side); and (**f**) dead plant from previous vintage.

Overall, we examined 5215 grape plants at the four grapevine germplasm collections (locations) combined. These plants represented 305 different cultivars. Many of the cultivars were present in more than one (up to four) location. Therefore, the number of samples analyzed was higher (537) than the number of cultivars (Table 2). GTD symptoms were categorized as new symptoms during the annual vegetative period (leaf stripes with white or brown rot and dieback) (Figure 1a–d) or as dead and missing (removed) plants from previous dieback events in past years (Figure 1e,f).

The disease incidence (DI%) was over 25% at each of the survey sites (Table 2); therefore, the conditions for a meaningful survey of GTD symptom expression were considered adequate for further analysis. The average proportion of plant loss within the GTD symptoms and disease incidence (i.e., all symptoms) was similar in each germplasm collection. Altogether, these results, with previous records of dieback symptoms of currently dead and removed (dead) plants, validated the connection between missing plants and previous dieback.

**Figure 2:**
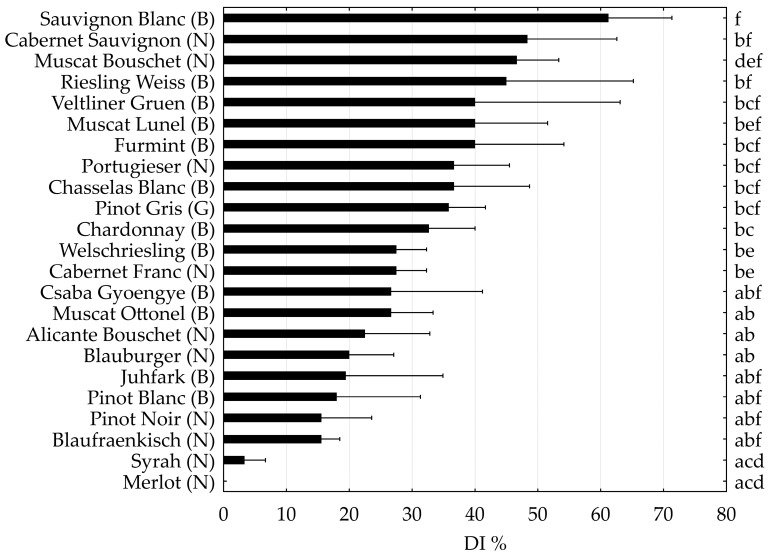
Disease incidence (DI) of grapevine trunk diseases of the most important international and national grape cultivars, surveyed in three or four Hungarian germplasm collections. The capital letters between brackets indicate the berry skin color: (N): noir, (B): blanc, and (G): gris, as defined in the VIVC database [51]. Small letters show significant differences based on the Mann–Whitney U-test (*p* < 0.05).

The DI of the most important cultivars with only *V. vinifera* ancestors were compared (Figure 2). Sauvignon Blanc and Cabernet Sauvignon were the most susceptible cultivars, while Merlot and Syrah were the less susceptible ones (Figure 2, Table 1). There were both white and red grapes among the most and the less sensitive cultivars within the genuine *V. vinifera* cultivars analyzed. The susceptibility of Furmint, one of the most important Hungarian white cultivars, was similar to that of Veltliner Gruen and Muscat Lunel, while another indigenous white cultivar, Juhfark, was less susceptible and more similar to that of Blauburger and Pinot Blanc. The indigenous table grape, Csaba Gyoengye, was less susceptible than Furmint, showing similar DI to those of Welschriesling, Cabernet Franc, and Muscat Ottonel. Blaufraenkish, a grapevine variety with regional importance was among the less susceptible cultivars, such as Pinot Blanc and Pinot Noir.

**Figure 3:**
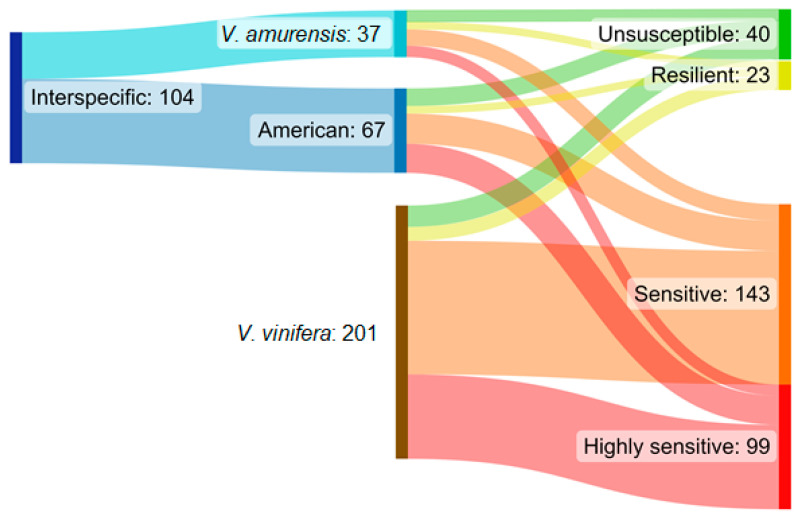
Distribution of the studied cultivars regarding their *Vitis* pure or mixed ancestry and the GTD pathogens sensitivity groups. Diagram created by SankeyMATIC [52].

The severity of the disease expression categories was defined as separate cultivars. When a cultivar tends to not demonstrate GTD symptoms in situ, it is defined as unsusceptible. When only annually developed (usually mild) GTD symptoms are displayed, the cultivar is listed as resilient. Sensitive cultivars demonstrated the tendency to develop dieback symptoms, eventually resulting in plant loss in parallel with other GTD symptoms in other individuals, while exclusively plant loss of infected specimens was detected in vulnerable cultivars that are highly sensitive. Most of the cultivars with only *V. vinifera* ancestors in their pedigree were categorized as highly sensitive or sensitive to GTDs with exclusive plant loss or high plant dieback concurrent with non-lethal symptoms (Figure 3). The level of resistance to GTD pathogens was generally better or much better in the case of interspecific hybrid *Vitis* cultivars, with a considerably higher ratio of unsusceptible or resilient cultivars than that encountered among the monophyletic *V. vinifera* ones (Figure 3).

**Figure 4:**
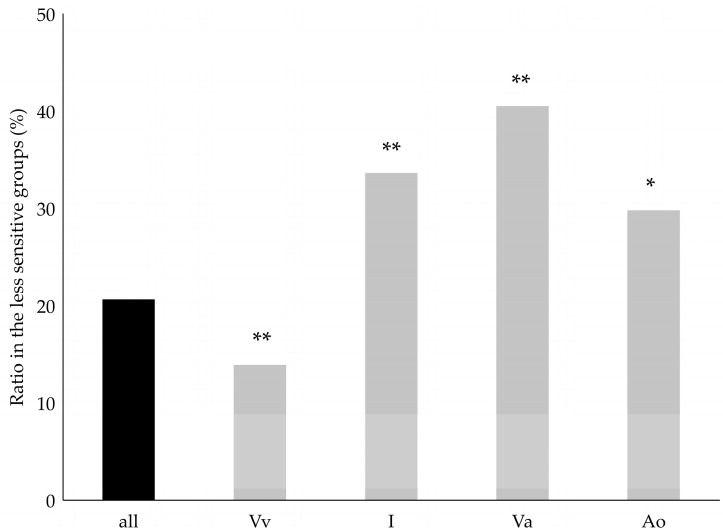
The ratio of the cultivars categorized in the less GTD pathogens sensitive group within monophyletic *V. vinifera* cultivars (Vv), and those in all interspecific cultivars combined (I), or with the interspecific cultivars with those with Asian (*V. amurensis*) (Va) or American species (*V. labrusca*, *V. riparia* or *V. rupestris*) (Va) ancestors treated separately. Results of the binomial probability test, indicating the difference between the examined group and the averages of all cultivars: * = *p* < 0.05; ** = *p* < 0.01.

The tendency of cultivars with different origins for plant loss was compared using a binomial test. The ratio of monophyletic *V. vinifera* cultivars was lower in the less sensitive groups (unsusceptible and resilient) than expected based on all tested cultivars (Figure 4). This indicates that monophyletic *V. vinifera* cultivars have a higher tendency to display serious GTD symptoms, including plant loss, than the average of all examined cultivars (overall samples). On the contrary, the ratio of cultivars without plant loss (less sensitive groups) was significantly higher in the group of interspecific hybrids. Similarly, when the hybrids with American (*V. labrusca*, *V. riparia*, or *V. rupestris*) or Asian (*V. amurensis*) ancestors were split and compared separately, the ratio of the cultivars in both groups was higher in the less susceptible categories compared to all the cultivars studied (Figure 4).

**Figure 5:**
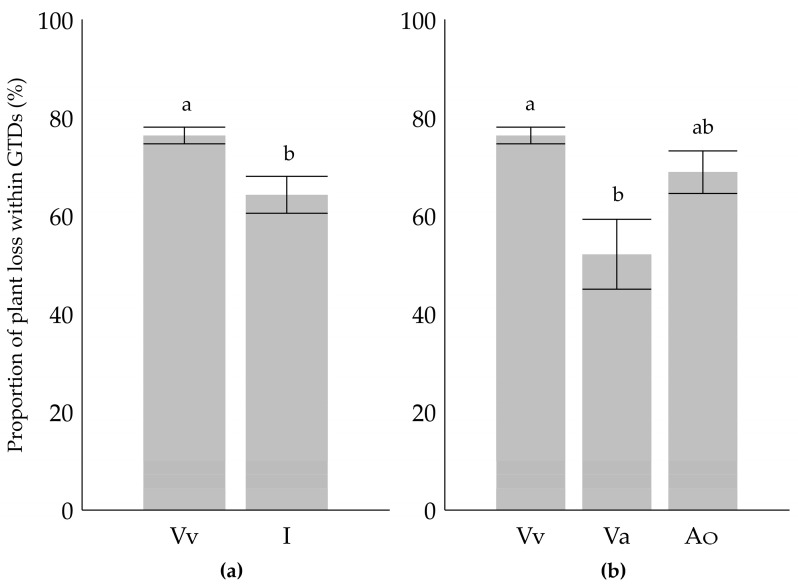
Proportion of plant loss within all recorded GTD symptoms (**a**) comparing the average of these cultivars with that in exclusively *V. vinifera* (Vv) ancestors, and with that in all the interspecific hybrids (I) and (**b**) the same comparison with Vv but now with hybrids with *V. amurensis* (Va) in their pedigree or those with American (Ao) (*V. labrusca*, *V. riparia* or *V. rupestris*) ancestry, separately. Small letters indicate significant differences between datasets based on the Mann–Whitney U-test (*p* < 0.01).

The susceptibility of cultivars with different species ancestry (i.e., exclusively *V. vinifera* or interspecific hybrids) was compared regarding the cultivar specimen mortality (proportion of plant loss) from GTDs as part of the GTD disease incidence (i.e., all symptoms). Plant death as a consequence of GTD expression was more likely in cultivars with a monophyletic *V. vinifera* origin than in the interspecific *Vitis* cultivars (Figure 5a). Separating the group of the interspecific cultivars into cultivars with Asian and American origins, the proportion of plant loss within the displayed GTD symptoms was meaningfully lower exclusively for cultivars with *V. amurensis* ancestry than the ones with monophyletic *V. vinifera* cultivars (Figure 5b). Thus, the calculated difference was not significant for the group of cultivars with *V. labrusca*, *V. riparia*, or *V. rupestris* (American species) in their pedigree.

## 3. Discussion

There are differences in sensitivity to GTDs displayed by *V. vinifera* cultivars; however, no completely resistant cultivars have been identified. The physiological and genetic background of these differences in sensitivity or resistance against GTD-causing pathogens is not understood [53,54]. In accordance with previous results, Sauvignon Blanc and Cabernet Sauvignon showed the highest DI in the surveyed Hungarian germplasm collections, all four with their own climate and soil characteristics, while Furmint, Chardonnay, and Cabernet Franc were found less GTD susceptible [12,42,43,46,55]. Blaufraenkisch (also referred to as Limberger), again confirmed by our current results, consistently had one of the lowest DI [42,43,46,55], while Merlot and Pinot Noir were usually also found to be less susceptible to most of GTDs in general [12,42,46,55,56].

Comparing the sensitivity of different grapevine cultivars to esca, significant differences were found between those with red and those with white berries, and their respective xylem vessel diameters and densities [46]. The average vessel diameter of the white cultivars was larger with higher densities, compared to the red grapevines. A similar trend was observed for the overall disease incidence, where the mean disease incidence was higher for white-berry cultivars than for red-berry cultivars. Foliar symptom symptoms are hypothesized to result from fungal toxins translocated to the leaves from primary infection sites [53,57,58]. Higher rates of leaf symptoms were explained by the larger vessel diameters, as they provide space for more intensive xylem cavitation, which can assist toxin translocation to the green plant parts [46]. Moreover, Pouzoulet et al. [59] stated that esca pathogens may escape compartmentalization more efficiently when the vessels are wider, and the more gel and tyloses in the vessels, the more substrate is provided for wood pathogens [59].

No GTD symptom expression was detected in the Hungarian germplasm collections of the Merlot cultivars, whose outstanding tolerance has been reported in several previous studies in other countries [21,39,42,44,50,60,61]. The lignin content of Merlot was found to be significantly higher than in Cabernet Sauvignon, a cultivar that is considerably more susceptible to GTDs [41,43,46,50]. Other cultivars identified as less sensitive to GTD had in general smaller vessel diameters and higher lignin content than the most sensitive grapevine varieties [62,63]. The results of Rolshausen et al. [62] highlighted the potential importance of lignin in the *E. lata*-grapevine interaction. The common defense response of grapevines to infection is compartmentalization, where the plant attempts to contain the invading agent by depositing suberin and lignin, which impedes the spread of pathogens throughout the xylem. A higher lignin content was detected in the infected grape tissues, which indicates that lignin deposition is initiated in response to fungal infection [62].

GTDs are complex diseases that result in serious economic losses by reduced grape productivity and are characterized by remarkable differences in disease severity and manifestation [2]. Infection with GTD fungal pathogens may result in latency, accidental or repeated annual disease expression, and serious partial or whole plant dieback [64]. The most serious disease symptom is plant loss, which can result in irreversible economic damage. Previously, only foliar or chronic and dead cordon or apoplectic (partial and whole plant) individual disease expressions were differentiated among the GTD symptoms [43,46,55]. This traditional categorization or subsequent merging of different symptom manifestations and calculating disease incidence indicates only the susceptibility of a cultivar and does not take into account the severity of the infection and the plant’s responses. Cultivars that are able to survive infection for a longer period of time–specimens that are more likely to express milder foliar symptoms and partial dieback rather than whole plant apoplexy and death—are considered more resistant to the fungal GTD pathogens in our present survey and analysis.

The survey and analysis of four Hungarian germplasm collections concluded that interspecific hybrid cultivars, in particular the ones with Asian *V. amurensis* ancestry, are generally less susceptible to GTDs, expressing no or milder symptoms, than monophyletic cultivars with only *V. vinifera* ancestors. In these hybrid cultivars with some level of East Asian ancestry, infection by GTD fungal pathogens resulted in less plant loss, which is the most serious and irreversible consequence of GTD infection. One of the possible backgrounds of this lower sensitivity (or higher resistance) may concur with the xylem vessel diameter, as *V. amurensis* had the smallest vessel diameter among the different grape species [65,66]. By contrast, the vessel diameter of the American species *V. labrusca* was reported to be rather large [65]. In a more recent study, there was no substantial difference in xylem vessel diameter recorded between *V. vinifera* and the American interspecific hybrid called Noiret with *V. labrusca* ancestry [63].

Since most of the GTD pathogens are wound-colonizing fungi, frost cracks in the wood parts of the plant could facilitate the prevalence of the GTD disease complex in grapevines [67,68]. Compared to *V. vinifera* and *V. labrusca* species, *V. amurensis* is extraordinarily cold resistant and can survive long and cold winters as a result of its relatively low respiratory intensity, lower level of active metabolism, and longer dormancy period [32]. *V. amurensis* is cultivated as a cold-resistant grape in the colder regions of China [32,69,70,71]. Wang et al. [72] identified 17 genes possibly involved in this increased cold hardiness. Accumulation of several amino acids (valine, isoleucine, and proline) was reported to be higher in *V. amurensis* than in *V. vinifera* cultivars, the level of which was subject to abiotic stress [73]. This property, together with the accumulation of other bioactive compounds (polyphenols, tannins, and stilbene phytoalexin resveratrol), can protect plants from long-term cold damage [32,74].

The induction of stilbene biosynthesis was correlated with basal immunity against downy mildew and eutypa dieback [48,75]. American *Vitis* species are also employed to breed more cold-hardy cultivars [76]. Increased stilbene biosynthesis has relevance in increased resistance to different fungal diseases [77] and may have importance in GTD tolerance, as grapevine rootstock transformed with grapevine stilbene synthase gene expressed from a pathogen-inducible promoter showed increased resistance against *E. lata* [48].

*V. amurensis* is not only cold-tolerant, but also resistant to white rot, grape anthracnose, and grape bitter rot (*Greeneria uvicola* (Berk. & M.A. Curtis) Punith) fungal diseases, and has a high resistance to downy mildew caused by the Oomycete *P. viticola* [29,31,32,33,74,78,79,80]. The resistance of grapevines against the bacterial trunk pathogen *A. vitis* was introgressed from *V. amurensis* upon interspecific breeding [30]. Hybrids with *V. amurensis* ancestry were unambiguously less sensitive to GTD pathogens in our survey, as illustrated by the considerably higher ratio of resilient and tolerant hybrid cultivars to Botryosphaeria dieback (BD) and esca diseases.

Pretorius and Høj [81] assumed that the product of a single gene or its pyramid (stacking multiple genes into a single genotype to combine desirable traits) is effective only against a narrowly related group of pathogens within the GTD complex. These authors differentiated tolerance toward various GTD pathogens in numerous monophyletic American *Vitis* cultivars and hybrids. The resistance loci Rda1 and Rda2 originating from *Vitis cinerea* (Engelm.) Engelm. ex Millard B9, a native American grape, and the interspecific Horizon cultivar, respectively, largely prevented the development of Phomopsis dieback symptoms [82]. Concordantly, an interspecific cultivar with parental varieties Catawba and *V. labrusca* showed reduced sensitivity to *Neofusicoccum parvum* (Pennycook & Samuels) Crous, Slippers & A.J.L. Phillips in inoculation assays. On the other hand, the American *Vitis* spp. were found to be more susceptible to Eutypa dieback than *V. vinifera* [44]. Co-evolution of *V. vinifera* and *E. lata* in a natural habitat could have increased the resistance of the plants prior to domestication [83].

One of the main goals of breeding programs nowadays is to pyramid extant, independent biotic and abiotic resistance genes from different lineages of American or Asian grapes and to attain additive accumulation of broad resistance against or tolerance to phytopathogens into one parent that can be crossed with European *V. vinifera* [84]. The domestication bottleneck effect, the result of thousands of years of vegetative propagation without meiosis and recombination, and the continuous incrossings of high-quality cultivars resulted in low genetic diversity across domesticated *V. vinifera* grapes [37,38]. Engaging American and Asian *Vitis* species in breeding has the potential to enhance biotic and abiotic vine stress tolerance lost over the course of domestication [29,30,31,32,33,34,35,36,69,70,71], which is relevant to GTD symptom expression and disease severity in grape cultivars.

## 4. Materials and Methods 

### 4.1. Survey Sites and Cultivars

**Figure 6:**
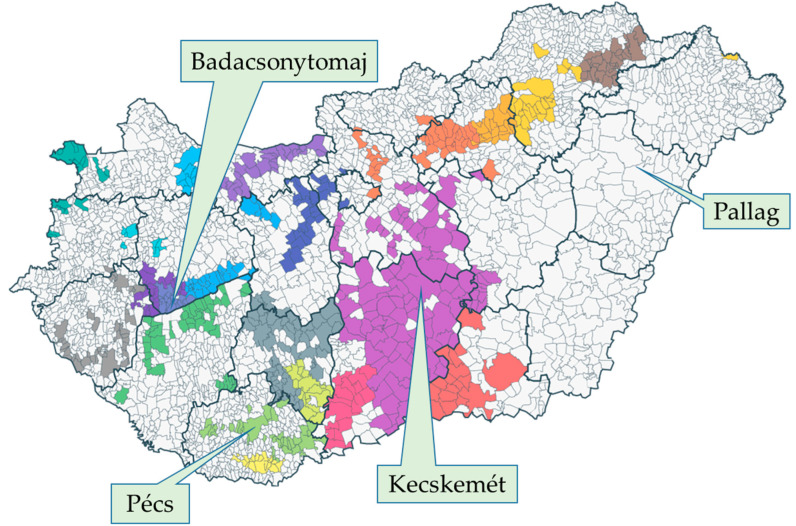
The location of the surveyed germplasm collections in Hungary. Different colors indicate different Wine Regions [87].

The survey was conducted in 2022, involving four Hungarian germplasm collections (Figure 6) containing a high number of cultivars with worldwide, Central-European, or Carpathian basin significance and valuable parental lines for further breeding. The climatic and edaphic conditions differed considerably at the four locations (Table 3), despite their geographical closeness (ranging from 60 to 330 km in distance). Pallag (University of Debrecen, Institutes for Agricultural Research and Educational Farm, Horticultural Experimental Plant of Pallag) and Kecskemét (Hungarian University of Agriculture and Life Sciences, Research Institute for Viticulture and Oenology) are in the eastern part of Hungary, which has a continental climate with relatively low annual precipitation (500–700 mm) [85]. These lowland sites in the Carpathian Basin were established on phylloxera immune sandy soils; thus, the plants growing at these locations were not grafted (Pallag) or in part growing on their own roots (Kecskemét) [86].

Badacsonytomaj (Hungarian University of Agriculture and Life Sciences, Research Institute for Viticulture and Oenology) and Pécs (University of Pécs, Research Institute for Viticulture and Oenology) are in the occidental part of the country, where the influence of westerly winds associated with a more moderate oceanic climate is more pronounced. Both of these sites have mountain slope relief with terrace cultivation and a sub-Mediterranean climate with annual precipitation between 600 and 800 mm [85,88]. The soil type in Badacsonytomaj is volcanic erubase and eroded loess slope sediment, and the region is heavily affected by the humidifying and moderating effects of the water body of the Lake Balaton [89]. The soil type in Pécs is Brown earth (Ramann’s brown forest soil) overlying carbonate-rich red sandstone.

**Table 3:** Characteristics of germplasm collection locations.

	Badacsonytomaj	Kecskemét	Pallag	Pécs
Climate	Submediterranean with dry, warm summer	Continental	Continental	Submediterranean with dry, warm summer
Soil ^1^	Erubase soil/Eutric Histosol	Sand/Haplic Arenosoil	Sand/ Haplic Arenosoil	Brown earth/Chromic Cambisol
Relief	Mountain slope (top-valley row direction, terrace cultivation)	Lowland	Lowland	Mountain slope (terrace cultivation)
Cultivation type	Grafted	Own rooted	Own rooted	Grafted
Relative climate sector ^2^	IIIc	Ib	Ia	IIIb
Average temperature fluctuation (°C)	21–22	23–24.5	23–24	21–22
Annual precipitation (mm)	600–800	500–550	550–700	600–800
Annual sunshine duration (h)	1950–2050	2000–2150	1900–2050	2000–2100

^1^ Soil types according to official Hungarian [90]/WRB [91] and European Commission [92]. ^2^ Relative climate sector as taken from [85].

The germplasm collections were considered to be free from the bacterial phytopathogens *A. vitis* and *Rhizobium radiobacter*. Vineyard parts potentially affected by the Flavescence dorée (*Ca*. *Phytoplasma vitis*) were consistently excluded from our survey. BD and esca symptoms were predominant at the surveyed sites, but Eutypa-like symptoms [13] were encountered in a few instances. The GTDs were visually diagnosed by the typical tiger-strip foliar symptoms (Figure 1a,d), while white and/or brown rot was detected on cross sections or debarked woody parts (Figure 1c) of the plants. The ensemble of BD, esca, and Eutypa-like symptoms was counted as GTD symptoms. The new apoplectic symptoms (dead young shoots with leaves, Figure 1b) were considered as annual GTD symptoms. If there were no fresh sprouts in the vine specimen, the plant was considered as dead (Figure 1e,f). All evaluated cultivars were surveyed in over 10-years-old plants, therefore the chronic/milder (non-lethal) symptoms were evaluable [93,94].

Many of the surveyed cultivars had non*-V. vinifera* ancestry. The different *Vitis* spp. in the pedigree of a cultivar were certified based on data from the Vitis International Variety Catalogue (VIVC) [51]. The cultivars were grouped for further analysis based on their ancestry from different *Vitis* spp. (Table 4). The parents of the interspecific cultivars are listed in Table S1.

### 4.2. Data Analysis

#### 4.2.1. Susceptibility Analysis

The disease incidence (DI%, the ratio of plants showing fresh leaf symptoms and dieback and whole plant apoplexy in previous years) was evaluated in the cultivars of the surveyed germplasm collections. Since the overall disease incidence was over 25% at every site and the spatial distribution of the symptom-expressing plants was homogenous in all vineyards, similar probabilities of infection were assumed for each cultivar. Given these conditions, the same cultivars in the different surveyed sites could be considered as replicates in the statistical analysis.

#### 4.2.2. Sensitivity Categories and Analysis

The cultivars were categorized based on a new method to determine the GTD disease expression severity (i.e., the severity of visible symptoms). Four categories were established to differentiate between: (1) no symptom expression, (2) exclusively new (annual) symptoms, (3) both new symptoms and previous dieback resulting in plant loss, and (4) exclusively previous dieback events all resulting in plant loss.

Four GTD sensitivity groups were created to categorize the studied cultivars based on the type (annual foliar symptoms and dieback or apoplexy) and the frequency of the different symptoms. Highly sensitive (HS), where all symptomatic plants of the cultivar are dead; sensitive (S), where both dead plants (resulting from apoplexy of the trunk) and fresh GTD leaves and dieback symptoms are detected. The cultivar was considered resilient (R) if only foliar symptoms were present, while neither apoplexy nor annual GTD leaf and dieback symptoms were detected in unsusceptible (U) cultivars (Table 5).

To reveal the potential differences in pathogen sensitivity among the different ancestry groups, the four original groups were re-appreciated, where the two more sensitive (HS and S) and the two less sensitive (R and U) categories were merged. The ratio of the lineage groups within each of these two redefined sensitivity categories was compared to the theoretically expected distributions with the binominal test.

The tendency of the GTD to kill the host plant was determined in parallel by calculating the proportion of individual plant losses within the disease incidence of the lineage groups and comparing the lineage groups in pairs. Monophyletic European *V. vinifera* (Vv) cultivars against the (1) interspecific (I) ones and (2) hybrids with American (*V. rupestris*, *V. riparia*, *V. labrusca*–Ao) and Asian (*V. amurensis*–Va) species co-origin.

### 4.3. Statistical Analysis and Software Background

The datasets did not fulfill the assumptions of parametric tests (i.e., normality and homogeneity of variances), which were analyzed with Q-Q plots and Levene’s test. During the analysis, the nonparametric Kruskal–Wallis test was used for comparison, which was backed up with the Mann–Whitney U-test for pairwise comparison with Statsoft Statistica ver.10 software.

The ratio of the sensitivity groups in different ancestral groups was compared with the binominal test executed using the online calculator of Stat Trek [95]. The Sankey diagram was generated by the Sankeymatic online diagram builder (https://sankeymatic.com, accessed on 18 January 2023).

## 5. Conclusions

Regarding the order in *V. vinifera* cultivar susceptibility based on disease incidence, earlier data from the literature in other grape-producing countries were confirmed, and the main cultivars of the Carpathian Basin were inserted in this ranking, where Juhfark proved to be more tolerant and Furmint more susceptible. Merlot did not show GTD symptoms in any of the Hungarian germplasm collections.

The interspecific *Vitis* cultivars had a lower tendency for plant loss following infection with GTD fungal pathogens. Hybrid varieties with Asian *V. amurensis* ancestry have outstanding tolerance in our experimental set of more than 300 cultivars. Engaging American and Asian *Vitis* species in breeding programs to enhance tolerance and resistance to GTDs has great potential.

## Figures and Tables

**Table 1:** The tolerance of *V. vinifera cultivars* to different grapevine trunk diseases. Adopted from Songy et al. [17].

	Cultivars ^1^	GTDs		Inoculation Test/Disease Incidence Survey ^3^	References
		**Tolerance**	**Disease ^2^**		
White	Chardonnay	high	BD, Eutypa	Test	[44]
		medium	BD	Survey	[43]
		medium	Esca	Survey	[41]
	Pinot Gris	high	BD, Eutypa	Survey	[43]
		medium	Esca	Survey	[10]
	Riesling	high	Eutypa	Survey	[43]
		medium	BD	Test	[45]
		medium/low	Esca	Test	[44]
	Sauvignon Blanc	high	BD	Test	[45]
		medium	Eutypa	Test and Survey	[43]
		low	BD	Test and Survey	[43]
		low	Esca	Survey	[42,46]
	Semillon	high	BD, Eutypa	Test and Survey	[43]
		low	Esca	Survey	[10]
	Thompson seedless	high	Esca	Test	[44,47]
		medium/low	Eutypa	Test	[44]
		low	BD, Eutypa	Test	[44,47]
	Ugni Blanc	medium/high	BD	Survey	[43]
		low	Eutypa	Test	[48]
		low	Esca, Eutypa	Test	[49]
				Survey	[41,43]
	Welshriesling	high	BD, Eutypa	Test and Survey	[43]
				Survey	[41]
		low	Esca	Survey	[7]
Red	Cabernet Franc	medium/high	Eutypa	Test	[44]
		medium	BD	Test	[44]
		low	Esca	Test and Survey	[43]
	Cabernet Sauvignon	high	BD	Test	[45]
		low	Eutypa	Test	[48]
		low	Esca, Eutypa	Survey	[41,46,50]
			BD	Survey	[43]
	Grenache	high	Esca, Eutypa	Survey	[43]
		high	Esca	Test	[47]
		BD	medium/high	Survey	[43]
	Merlot	high	Eutypa	Test	[44,48]
		medium/high	BD	Test	[44]
		medium	Esca	Survey	[42,50]
	Pinot Noir	high	Esca	Survey	[41]
			Eutypa, Esca	Test and Survey	[43]
		medium	BD	Test and Survey	[43]
	Sangiovese	high	BD, Esca, Eutypa	Test and Survey	[43]
		medium	Esca	Survey	[41]
	Syrah	high	Esca	Survey	[41]
		low	BD, Eutypa	Test	[21,44]
				Test and Survey	[43]
Hybrid (*V. labrusca* hybrid)	Concord (*Vitis labrusca* hybrid)	high	BD, Esca, Eutypa	Test	[44]

^1^ Cultivar primer names from VIVC database [51]. ^2^ BD: Botryosphaeria dieback; Eutypa: Eutypa dieback. ^3^ Test: Inoculation of cuttings; Survey: in field survey of disease incidence.

**Table 2:** Disease incidence (DI) of grapevine trunk diseases (GTDs) (mean ± SE) and the proportion of plant loss (mean ± SE) within GTD symptoms in different germplasm collections. Small letters show significant differences based on the Mann–Whitney U-test (*p* < 0.05).

Location	No. Samples *	GTDs	
		DI% (±SE)	Proportion of Plant Loss (% ± SE)
Badacsonytomaj	90	44.58 (±2.62) c	74.63 (±3.14) a
Kecskemét	130	28.05 (±2.19) a	76.49 (±3.11) a
Pallag	166	37.05 (±2.16) b	73.78 (±3.10) a
Pécs	151	28.41 (±1.92) a	69.94 (±3.23) a
Total	537	33.70 (±1.13)	73.56 (±1.59)

* In the case of locations, the number of samples are equal to the number of cultivars.

**Table 4:** Categories of cultivars with multiple *Vitis* species ancestry.

Ancestors in Parent or Grandparent Level	Categorization I.	Categorization II.
*Vitis vinifera*	*Vitis vinifera* (Vv)	*Vitis vinifera* (Vv)
Occurrence of American species ^1^	Interspecific (I)	American origin (Ao)
Occurrence of *Vitis amurensis*	Interspecific (I)	*Vitis amurensis* origin (Va)

^1^*V. labrusca*, *V. riparia* or *V. rupestris*.

**Table 5:** Categorization of cultivars according to the observed sensitivity toward grapevine trunk diseases (GTD).

Sensitivity Categories		GTD Symptoms	
Two Groups	Four Groups	Apoplexy (Dead Plant)	Leaf Symptoms and Fresh Dieback
More sensitive	Highly sensitive (HS)	Exclusively	-
	Sensitive (S)	Present	Present
Less sensitive	Resilient (R)	-	Exclusively
	Unsusceptible (U)	-	-

## Data Availability

The datasets used in the current study are available from the corresponding author on reasonable request.

## Supplementary Materials

The following supporting information can be downloaded at: https://www.mdpi.com/article/10.3390/plants12122328/s1. Table S1: Parents of the surveyed interspecific *Vitis* cultivars.
