# Supplementary material for: Hybrid Vitis Cultivars with American or Asian Ancestries Show Higher Tolerance towards Grapevine Trunk Diseases
Source: Plants (Basel). 2023 Jun 15;12(12):2328. doi: 10.3390/plants12122328 (PMC10305513; doi:10.3390/plants12122328)
Supplement: Supplementary file 1 [file plants-12-02328-s001.zip › plants-2378490-supplementary.pdf]

Table S1. Parents of the surveyed interspecific *Vitis* cultivars.

Cultivar name in the germplasm collection	Parent 1	Parent 2
Interspecific cultivars with American origin		
Aletta	Muscat Ottonel	Villard Blanc
Angela	Villard Blanc	Halili Krasznuey
Aron	Villard Blanc	Perlette
Baco-1	Folle Blanche	Riparia Grand Glabre + V. riparia
Bianca	Villard Blanc	Bouvier
Concord	Catawba	*Vitis labrusca*
Couderc	Munson	Raisaine
Csillam	Rayon D'or	Blaufraenkisch
Dalmadin	Catawba	*V. labrusca*
Duna Gyongye	Seibel 4986 X Csaba gyoengye	SEIBEL 4986 + ?
Elvira	Taylor	Martha
Esther	Villard Blanc	Magaracsi csemege
Eureka	America	Ontario
Fanny	Villard Blanc	Parent 2 was not confirmed
Fehér Delaware	*V. labrusca*	*Vitis vinifera* subsp. *vinifera*
Fenyeslevelu Izabella	*V. labrusca*	Meslier Petit
Flora	*V. vinifera* subsp. *vinifera*	*V. labrusca*
GM. 318-57	Chancellor	Riesling
GM. 7116-26	Geisenheim 323-58	Ehrenfelser
GM. 7743-15	Riesling	Geisenheim 6495- 1
Gocseji zamatos	Seyve Villard 12- 286	Medoc Noir
Hibernal (GM. 322-58)	Seibel 7053 + Riesling	?
Jazmin	Bianca	Petra
Kék Izabella	*V. labrusca*	Meslier Petit
Lakhegyi mezes	Honigler	Villard Blanc
Lidi	Villard Blanc	Magaracsi csemege
Lilla	Villard Blanc	Pannonia kincse x Mathiasz Janos diadala
Medina	Seyve Villard 12- 286	Medoc Noir
Moldova	Guzal Kara	Villard Blanc
Nero	Villard Blanc	Gardonyi Geza
Othello	Munson	*V. vinifera* subsp. *vinifera*
Palatina	Koenigin der Weingaerten	Villard Blanc
Piros Delaware	Labrusca x aestivalis	V. vinifera
Poloskei muskotaly	Zala gyoengye	Erzsebet kiralyne emleke
Reflex	Pannonia kincse	Aurore
Reform	Csaba gyoengye	Aurore
Refren	Gloria Hungariae	Aurore
Regent	Diana	Chambourcin
RF 16	Gloria Hungariae	Aurore
RF-38/32	Seibel 5279	*V. vinifera*
Seibel 1	Munson	*V. vinifera* subsp. *vinifera*
Seibel 11803 (Rubilande)	Bienvenu	Roi des Noirs
Seibel 13053 (Cascade)	Seibel 7042	Seibel 5409
Seibel 2	Munson	*V. vinifera* subsp. *vinifera*
Seibel 4643 (Roi des Noirs	Seibel 29	Dangue
Seibel 4646 (Pourpre)	Seibel 2508	Seibel 880
Seibel 4986 (Rayon D'or)	Seibel 405	Seibel 2007
Seibel 5409 (Glorie de Seibel)	Seibel 867	Seibel 452

Seibel 5450	Seibel 867	Seibel 4182
Seibel 5455 (Plantet)	Seibel 867	Seibel 2524
Seibel 7053 (Chancellor)	Seibel 5163	Seibel 880
Seibel 8718	Seibel 5163	Seibel 880
Seibel 8745 (Seinoir)	Seibel 5163	Seibel 880
Seyve Villard 12286	Subereux	Seibel 6468
Seyve Villard 12303	Subereux	Seibel 6468
Seyve Villard 12358	Subereux	Seibel 6468
Seyve Villard 12364	Subereux	Seibel 6468
Seyve Villard 12375 (Villard Blanc)	Subereux	Seibel 6468
Seyve Villard 12390	Subereux	Seibel 6468
Seyve Villard 18315 (Villard Noir)	Chancellor	Subereux
Seyve Villard 20365 (Dattier de St. Vallier)	Muscat Hamburg	Villard Blanc
Seyve Villard 20473 (Muscat de St. Vallier Blanc)	Seyve Villard 12129	Muscat Hamburg
Seyve Villard 23657 (Varousset)	Seibel 4668	Subereux
Seyve Villard 5276 (Seyval Blanc)	Seibel 5656	Rayon D'or
Suzy	Villard Blanc	Pannonia kincse
Terez	Villard Blanc	Olimpia
Zala gyoengye	Roucaneuf	Csaba gyoengye
Interspecific cultivars with Asian (*V. amurensis*) origin		
A 15-10/1	*Vitis amurensis* ancestor. Pers. com. Dr. Pál Kozma breeder. kozma.pal@pte.hu	
A 15-11-6		
A 15-13/4		
A 15-7-1		
A 15-8/3		
A 5-10-6		
A 5-11/2		
A 5-11/6		
A 5-14/5		
A 5-1-5		
A 5-16-3		
A 5-18-4		
A 5-21/1		
A 5-23/5		
A 5-4-6		
Alfold-100	Thalloczy Lajos	*V. amurensis* x *V. vinifera*
Amadeus	Chardonnay	*V. amurensis* x *V. vinifera*
Bruszkam	Bruskovatenkii	*V. amurensis*
Kaberam	Cabernet Sauvignon	*V. amurensis*
Korai bibor	*V. amurensis* x *V. vinifera*	Irsai Oliver
Kunbarat	Koleda 28/19	Afus Ali
Kunleany	Koleda 28/19	Afus Ali
Odysseus	(*V. amurensis* x *V. vinifera*) X Thalloczy Lajos Muskotaly	Pinot Gris
Orpheus	*V. amurensis* x *V. vinifera*	Irsai Oliver
Pannon frankos	(*V. amurensis* X *V. vinifera*) F2	Irsai Oliver
SK 77/11/87	Kunleany	Traminer
Panonija (SK 90-2/19)	Sremski Karlovci 86 2-239	Riesling
SK 77-10/53	Welshriesling	Kunbarat
SK 77-10/54 (Lela)	Welshriesling	Kunbarat

SK 77-14/17 (Mila)	Kunleany	Muscat Ottonel
SK 77-2/10	Kunleany	Traminer
SK 77-3/16	Saperavi Severny	Blaufraenkisch
SK 77-3/9	Saperavi Severny	Blaufraenkisch
SK 77-4/4	Kunbarat	Traminer
SK 77-4/5	Kunbarat	Traminer
Vosztorg	Zarya Severa x Dolores	Ruskii Rannii
Zarya Severa	Seyanets Malengra	*V. amurensis* x *V. vinifera*
